# Testis specific Y-like 5: gene expression, methylation and implications for drug sensitivity in prostate carcinoma

**DOI:** 10.1186/s12885-017-3134-7

**Published:** 2017-02-24

**Authors:** Senthil R. Kumar, Jeffrey N. Bryan, Magda Esebua, James Amos-Landgraf, Tanner J. May

**Affiliations:** 1 Comparative Oncology Radiobiology and Epigenetics Laboratory, College of Veterinary Medicine and Surgery, University of Missouri, 1600 E Rollins, W-143 Veterinary Medicine Building, Columbia, MO 65211 USA; 2 Department of Pathology and Anatomical Sciences, School of Medicine, University of Missouri, Columbia, MO 65212 USA; 3 Department of Veterinary Pathobiology, University of Missouri, Columbia, MO 65211 USA

**Keywords:** Prostate carcinoma, Putative tumor suppressor gene, Drug sensitivity, Methylation

## Abstract

**Background:**

TSPYL5, a putative tumor suppressor gene, belongs to the nucleosome assembly protein family. The chromosomal location of the *TSPYL5* gene is 8Q22.1, and its exact role in prostate cancer etiology remains unclear. Further TSPYL5 gene and protein expression in prostate carcinoma cells and diseased tissues including its susceptibility for epigenetic silencing is unknown. Also, not known is the variation in TSPYL5 protein expression with regards to progression of prostatic carcinoma and its possible role in drug sensitivity.

**Methods:**

*TSPYL5, DNMT-1* and *DNMT-B* gene expression in DU145, LNCaP and RWPE-1 cells and prostate tumor tissues was analyzed by qRT-PCR and RT-PCR. Demethylation experiments were done by treating DU145 and LNCaP cells with 5-aza-2′-deoxycytidine in vitro. Methylation analysis of *TSPYL5* gene was performed by methylation specific PCR and pyrosequencing. TSPYL5 protein expression in benign and diseased prostate tumor tissues was performed by immunohistochemistry and in the cells by Western blotting.

**Results:**

*TSPYL5* was differentially expressed in non-tumorigenic prostate epithelial cells (RWPE-1), androgen independent (DU145), dependent (LNCaP) prostate carcinoma cells and tissues. Methylation-specific PCR and pyrosequencing analysis identified an inverse relationship between DNA methylation and expression leading to the silencing of *TSPYL5* gene. Treatment of prostate carcinoma cells in which TSPYL5 was absent or low (DU145 and LNCaP) with the demethylating agent 5-aza-2′-deoxycytidine upregulated its expression in these cells. Immunohistochemical studies clearly identified TSPYL5 protein in benign tissue and in tumors with Gleason score (GS) of 6 and 7. TSPYL5 protein levels were very low in tumors of GS ≥ 8. TSPYL5 overexpression in LNCaP cells increased the cell sensitivity to chemotherapy drugs such as docetaxel and paclitaxel, as measured by the cellular viability. Furthermore, the cells also exhibited reduced CDKN1A expression with only marginal reduction in pAKT.

**Conclusions:**

Decrease in TSPYL5 protein in advanced tumors might possibly function as an indicator of prostate tumor progression. Its absence due to methylation-induced silencing can lead to reduced drug sensitivity in prostate carcinoma.

**Electronic supplementary material:**

The online version of this article (doi:10.1186/s12885-017-3134-7) contains supplementary material, which is available to authorized users.

## Background

Prostate cancer remains a major public health problem in developed countries with an estimated 181,000 new cases in 2016 in the United States [1]. The disease can progress from a hormone sensitive to castrate-resistant phenotype and eventually metastasize [2]. Multiple factors, including screening using prostate specific antigen (PSA) levels, and an aging population have resulted in increased frequency of diagnosis of early stage prostate tumors, most of which do not require immediate therapeutic intervention [3]. However, a small number of high-grade tumors are underdiagnosed and undertreated.

Therapies for cancer including that of the prostate have shifted from administering broadly acting cytotoxic drugs to specific therapies targeted to each tumor. In order to facilitate the shift, a “precision-medicine” approach where tests that predict the clinical outcome of patients on the basis of genes expressed by their tumors are likely to influence patient management and drug development. Molecular signatures will have utility both in clinical management of disease and in elucidating the mechanism involved, thereby providing insight into potentially novel therapies [4–6].

Testis specific Y-like-5 (*TSPYL5*, *KIAA1750*) is a member of testis-specific protein Y-encoded-like (TSPY-L) family of genes, whose functions are currently unknown [7]. Testis specific Y-like (TSPYL) proteins are members of the nucleosome assembly protein (NAP) superfamily [8]. TSPYL proteins show high sequence homology to NAP’s which possess a highly conserved NAP domain (~180 amino acids) that participates in histone binding. In general the NAP proteins participate in transcriptional regulation [9] and in regulation of the cell cycle [10]. Also, NAP-1 shuttles histones between the cytoplasm and nucleus, assembles nucleosomes and affects transcription of many genes by promoting chromatin fluidity [11].

Silencing of tumor suppressor genes (TSG’s) by aberrant DNA methylation at critical gene control regions plays a central role in the development of cancers [12]. Alternately, a decrease in methylation at specific sequences could increase the expression of cancer-promoting genes [13]. The *TSPYL5* gene is of particular interest because, apart from the documented role as a putative TSG in glioblastoma and gastric cancer [7, 14], it has been implicated in cancer signaling pathways involving CDKN1A (p21, WAF1/Cip 1) and pAKT in lung carcinoma cells [15]. CDKN1A has been implicated in both anti-proliferative, pro-proliferative and survival roles [16]. Moreover, AKT activation increases cell survival and proliferation [17]. It is likely that TSPYL5 could participate in more than one function, depending on the cell type and its epigenetic modulation. Overall, little is known about the definite role of this gene in carcinomas including that of the prostate.

It is hypothesized that more advanced prostate tumors will have low *TSPYL5* gene and protein expression compared to moderately advanced or normal phenotype, and such differential expression of TSPYL5 is due to epigenetic modulation of this gene. To gain insight into the role of *TSPYL5* in prostate cancer, we investigated its expression, methylation pattern, its role in signaling pathways and drug sensitivity and presence of its protein with respect to disease severity. In this study we report that *TSPYL5* gene and protein expression varied in prostate adenocarcinoma (PC) cells and human benign and prostate tumor tissues as analyzed by qRT-PCR and immunoblotting. Consistent with variable TSPYL5 expression in cells and tissues, more advanced tumor tissues had an inverse correlation between methylation and gene or protein expression as studied by methyl-specific PCR (MSP), pyrosequencing (PSQ) and immunohistochemistry (IHC) analysis. We also report that in low TSPYL5 protein expressing PC cells, varied expression of proteins such as pAKT was observed. Moreover, TSPYL5 may play a role in sensitivity to chemotherapy likely by modulating pleiotropic protein such as CDKN1A.

## Methods

### Chemicals and antibodies

Demethylating agent 5-aza-2′-deoxycytidine (Decitabine, DT) was from (Sigma Chemical Company, St Louis, MO). Antibodies used were rabbit anti-TSPYL5 (Immunoblot), rabbit anti- CDKN1A (Thr-145) (Santa Cruz Biotechnology. Santa Cruz, CA), rabbit anti-TSPYL5 (Sigma, Immunohistochemistry), rabbit anti-AKT, mouse anti-DNMT3B (Novus Biologicals, Littleton, CO), anti-DNMT1, anti-PTEN, anti-β-actin, anti-Histone-H3, anti- p-CDKN1A (T-145), anti-pAKT (Ser- 473) (rabbit), including secondary HRP-conjugated anti-rabbit and mouse (Cell Signaling Technology, Danvers, MA). Chemotherapy drugs paclitaxel (px) and docetaxel (dtx) were procured from the local veterinary pharmacy.

### Cells and patient tumor specimens

The PC cell lines, DU145, LNCaP and non-tumorigenic (NT) prostate epithelial cells RWPE-1 were purchased from ATCC (Manassas, VA). All of the carcinoma cells were maintained in custom RPMI or DMEM/F12 media with 10% FBS and Gentamycin. The RWPE-1 cells were maintained in a keratinocyte serum free media with growth factor supplements. The cells were tested routinely for mycoplasma contamination with the MycoAlert luciferase kit (Lonza, Allendale, NJ). Archival formalin fixed paraffin embedded (FFPE) tumor specimens from normal, benign or prostate carcinoma patients were obtained from the Pathology department at the University of Missouri Hospital after institutional IRB approval.

### Demethylation of *TSPYL5* in PC cells

The PC cells DU145 and LNCaP were treated with a demethylation drug DT (0.5 μM) for 4 days with fresh addition of DT every 12 h. Subsequently, total RNA was isolated and reverse transcribed to cDNA. qRT-PCR was performed to analyze *TSPYL5* gene expression in drug treated and untreated samples.

### cDNA synthesis and PCR amplification

Total RNA from prostate carcinoma cells (DU145 and LNCaP), epithelial cells (RWPE-1) and FFPE prostate tissues was extracted using RNeasy or RNeasy FFPE kits (Qiagen, Valencia, CA), respectively. cDNA was generated from total RNA using a cDNA synthesis kit (Bio-rad, Hercules, CA). PCR was performed with *TSPYL5* primers. β-*actin* was used as a housekeeping gene. The PCR conditions were as follows: denaturation at 98 °C for 1 min, followed by 28 cycles at 95 °C for 30 s, 55 °C for 30 s and 70 °C for 30 s, with a final extension at 70 °C for 8 min. The amplified PCR products were analyzed by 2% agarose gel electrophoresis containing Gel Red (Biotium, Hayward, CA). Quantitative real-time PCR (qRT-PCR), was performed with CFX Connect and a Sybr Green reaction (Biorad). The following primers were used for *TSPYL5* PCR: Forward, 5′-TGGGCCCTTCTACTGGTGAACTTT-3′; Reverse, 5′- TCACCTGGAGCCACAGCATAATGA-3′. The mRNA expression in tissues was analyzed and the relative cumulative density was calculated by measuring area under curve (AUC) for each sample using an image processing and analysis program (Image J, NIH). Percentage average was obtained for each group and an arbitrary number of 1 was assigned for highest percentage group and subsequent groups were assigned numbers relative to 1 for graphical representation.

### Genomic DNA isolation and bisulfite conversion

The genomic DNA isolated from PC cells using DNeasy Blood & Tissue Kit or tumor tissues using QIAamp DNA FFPE Tissue Kit (Qiagen) was bisulfite-modified with EZ-DNA Methylation-Gold Kit (Zymo Research, Irvine, CA) according to manufacturer’s instructions. The bisulfite reaction was carried out with 500 ng genomic DNA. Bisulfite converted DNA samples were stored at −20 °C until further use.

### Methyl specific PCR and pyrosequencing analysis

Methyl specific PCR (MSP) was performed in PC cells as well as FFPE tumor tissues using bisulfite-converted DNA with primers designed to include two CpG dinucleotides in each forward and reverse primer. Two sets of primers (CpG island) were designed; one, for methylated sequence (which retains CpG complementarity); 5′-GAGGTTATAGTTTAGGGGGAGTTG-3′; R- 5′- CCAAACAACACAAATACAAACTAAC-3′. For unmethylated sequences (complimentary to TpG sequence), the primers F- 5′-GAGAAATTTGTTGAGATTTAAAGTGA-3′; R- 5′CCATCACAAAAAAACATAATA-CACC-3′ were used. The presence of a methylated band in PCR is indicative of methylation in the original sequence [18]. Primers were designed using MethPrimer program [19]. The MSP and unmethylated sequence (USP) PCR bands in tissues upon gel electrophoresis (2% agarose) were analyzed for AUC using the Image J program. The percent methylation for each sample was calculated using AUC of methylated A (M) and unmethylated bands A (U) as follows: Percentage = A (M) ×100/A (M) + A (U).

Pyrosequencing (PSQ) of genomic DNA to quantitate the methylation of individual cytosine residues was performed as described earlier [20]. PSQ is a fast, reliable and quantitative method for analysis of CpG methylation [21]. Methylation analysis of DU145, LNCaP and RWPE-1 cells was performed with *TSPYL5* specific primers (CpG island shores) which consisted of a forward (5′- AGAGAAAGTAAAGGTGGATGTTATAATGT-3′), biotinylated reverse (5′-Biosg/ATACTTCCATCCCTTACTATATAACCTA-3′) and sequencing primers (5′-AAAGGAGGTGTTGATAT-3′) designed for a *TSPYL5* promoter sequence, followed by DNA sequencing in a Pyro Mark ID system by employing the Pyro Gold reagents kit (Life Technologies, Grand Island, NY). The primers were designed using a PSQ assay design program. The average degree of methylation at four CpG sites was analyzed using Pyro Mark ID software and results are depicted as percentage methylation.

### Patient samples and IHC analyses

IHC studies were performed as described previously [22] to identify the protein expression levels and cellular localization of TSPYL5 in non-malignant and malignant FFPE human prostate tissues using intelliPATH FLX (Biocare Medical). The analyzed tissue specimens included core tissue from patients with prostate adenocarcinoma (Gleason scores (GS) ranging from 6 to 9), normal and benign prostate tissues. Human testis tissue was used as positive control to detect TSPYL5 protein expression.

Immunoreactivity was scored by a board-certified pathologist (ME) in at least five random fields at 400× magnification in each section and the intensity of protein staining was scored on a 0–3+ scale (0 = no staining, 1 + = weak staining, 2 + = moderate staining, and 3 + = strong staining). The percentage of cells staining positive was scored on 1 - 4 scale (1 = 0–25% positive PC cells, 2 = 26–50% positive cells, 3 = 51–75% positive cells, and 4 = 76–100% positive cells). Composite score (CS) (0–12) was obtained by multiplying the staining intensity and percent of immunoreactive cells. Statistical significance was evaluated by the Mann–Whitney test. *P* < 0.05 was considered significant. H & E staining was performed according to standard procedures described in literature. Grading is assigned according to 2005 International Society of Urological Pathology Consensus Statement on Gleason Grading of Prostate Cancer (Epstein JI, Allsbrook WC Jr, Amin MB, Egevad LL; ISUP Grading Committee. The 2005 International Society of Urological Pathology (ISUP) Consensus Conference on Gleason Grading of Prostatic Carcinoma [23].

### TSPYL5 overexpression in LNCaP cells

For overexpression of TSPYL5, LNCaP cells were plated in 6 well plates (0.3-1 ×10^6^/well) and allowed to grow to 70–80% confluency at 37 °C. The mammalian expression vector TSPYL5/pCMV6-AN-GFP (PV-TSPYL5) or pCMV6-AN-GFP (PV) (Origene, Rockville, MD) with N-terminal tGFP tag was transiently transfected into LNCaP cells using Lipofectamine 3000 (ThermoFisher Scientific, Walthem, MA) according to the manufacturer’s protocol and were allowed to grow for 72 h, harvested and subsequently used for further studies.

### Cell viability

A cell viability assay was performed as described previously [24] using a WST-1 assay (Roche Applied Science, Indianapolis, IN) with or without 10 nM of chemotherapy drugs px or dtx. The results are expressed as percent viable cells after respective analysis. All experiments were performed in triplicate.

### Immunoblotting

Protein was extracted from whole cell lysates using the M-PER mammalian protein extraction reagent (Thermo Scientific), and the concentrations were estimated by the Bradford method. Equal amounts of protein (35 μg) were loaded on to the gel. Subsequently, the proteins were blotted on to a nitrocellulose membrane. The membrane was probed separately with primary antibodies for TSPYL5, CDKN1A, and AKT including P-CDKN1A (Thr 145), pAKT (S-473), β-actin, histone H3, PTEN, DNMT-1 and DNMT3b. Following incubation with the primary antibody at 4 °C overnight, the membrane was incubated with a horseradish peroxidase-labeled secondary antibody and visualized with Luminate Forte Western HRP substrate (Millipore, Billerica, MA). The blot was imaged in a Kodak imaging station (Carestream Health). The protein band ratios were calculated from the protein band intensities obtained using Image J program.

### Statistical analysis

Independent experiments were performed a minimum of three times. Statistical analyses on experiments were performed by unpaired two-tailed Student’s t-test for protein expression evaluations, one-way analysis of variance (ANOVA) for RT-PCR and Mann–Whitney U test for immunohistochemical analysis. The graphs were generated using GraphPad Prism 6 (GraphPad Software Inc., San Diego, CA). *P* ≤ 0.05 was considered significant.

## Results

### TSPYL5 gene and protein was variably expressed in prostate carcinoma and NT prostate epithelial cells

**Fig. 1:**
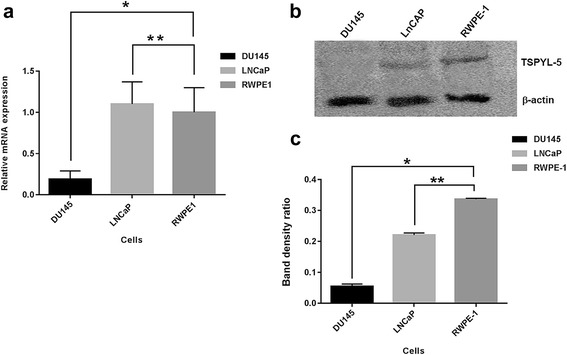
*TSPYL5* mRNA expression in non-tumorigenic and PC cells. **a** qRT-PCR analysis for *TSPYL5* mRNA showing relative expression between non- tumorigenic and PC cells (mean ± SD, *n* = 3). TSPYL5 mRNA was low in DU145 cells compared to RWPE1 (*P* = 0.02^*^), while no significant change was observed between LNCaP and RWPE1 cells (*P* > 0.05^**^). The values were normalized against β-actin. **b** Immunoblot analysis for TSPYL5 protein (~48 kDa) and β-actin (~46 kDA) expression in total cell lysates after chemiluminescent detection of bands. **c** The band intensity ratio of TSPYL5 was analyzed using Image J program. Decrease in TSPYL5 protein expression was significant between RWPE-1 and DU145 (*P* = 0.001^*^) and moderate between RWPE-1 and LNCaP (*P* = 0.04^**^) cells. All the values were calculated after subtracting the background intensity. Analysis was done in three repeat individual experiments

*TSPYL5* gene expression was analyzed in triplicate in PC cells (DU145, and LNCaP) and non-tumor (NT) epithelial cells (RWPE-1) by qRT-PCR analysis. qRT-PCR analysis indicated variable *TSPYL5* mRNA expression in the cells tested (Fig. 1a). While the TSPYL5 mRNA expression was not significant between RWPE-1 and LNCaP (P ≥ 0.05), the expression was significantly low in DU145 cells (*P* = 0.02). Total cell lysates were analyzed for TSPYL5 protein expression by immunoblot analysis. As anticipated, the protein expression was very insignificant in DU145 but low to moderate in LNCaP and RWPE-1 cells (Fig. 1b), respectively. The difference in TSPYL5 protein expression was evaluated based on protein band intensities (Fig. 1c). The decrease in TSPYL5 protein expression was found to be highly significant between RWPE-1 and DU145 (*P* = 0.001) while moderate difference was observed between RWPE-1 and LNCaP, (*P* = 0.04) cells.

### *TSPYL5* gene is methylated and responds to demethylation drug DT

**Fig. 2:**
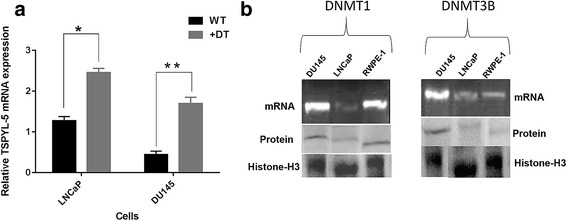
Reactivation of *TSPYL5* mRNA and expression of DNMT’s in PC cells. **a** Induction of *TSPYL5* mRNA expression in DU145 and LNCaP cells upon treatment with DT (0.5 μM) for 4 days at 37 °C. Significant induction of *TSPYL5* mRNA was observed both in LNCaP (*P* = 0.001^*^) and DU145 cells (*P* = 0.0021^**^). **b** RT-PCR for *DNMT1* and *DNMT3B* mRNA and immunoblot analysis for protein expression in nuclear fractions of the PC cells. Histone-H3 was used as a housekeeping protein

Due to the differential expression of mRNA in various cells used in this study, we analyzed the *TSPYL5* gene methylation status in all the cells. First, we treated endogenously absent or low *TSPYL5* expressing PC cells DU145 and LNCaP with DT. DT was effective in strong induction of *TSPYL5* mRNA in LNCaP (*P* = 0.001) and DU145 (*P* = 0.0021) cells compared to wild type (WT) counterparts (Fig. 2a), suggesting that *TSPYL5* gene is a target primarily for aberrant methylation. Next, in order to analyze the presence of DNA methylating enzymes DNMT1 and DNMT3B, an RT- PCR was performed to observe the variation in mRNA of these enzymes across the cells tested. As shown (Fig. 2b), all the cells had mRNA expression of these enzymes. Interestingly, it also was evident in DU145 cells where endogenous *TSPYL5* expression was very low, both *DNMT1* and *DNMT3b* mRNA expression was relatively high compared to the other cells, in which one or the other of the enzyme mRNA expression was low. Varying DNMT1 and DNMT3B protein expression was also observed in the nuclear fraction of the cells. Histone–H3 was used as a housekeeping protein.

**Fig. 3:**
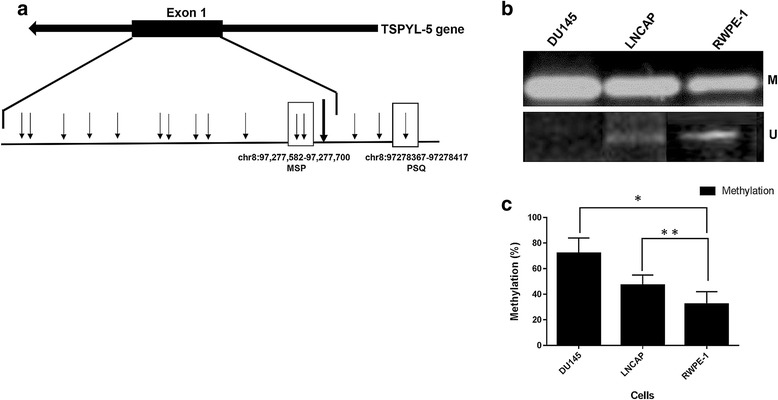
MSP analysis in PC cells. **a** CpG plot of the 5′-regulatory regions of *TSPYL5* gene (*small arrows* denote CpGs) and *large arrow* denotes transcription start site. The *box* represents the location of chromosomal 8 region analyzed by MSP or PSQ. **b** MSP reactions with methylated DNA-specific primers (M), and unmethylated DNA-specific primers (U). **c** Methylation was significantly higher in DU145 (*P* =0.001^*^) and LNCaP (*P* = 0.012^**^) cells compared to RWPE-1. Unmethylated bands was present in both RWPE-1 and LNCaP cells but absent in DU145 cells. The PCR reactions was performed in three repeat individual experiments

We further investigated the *TSPYL5* gene methylation status by MSP analysis. The MSP primers were designed within the chromosomal 8 regions (97,277,582-97,277,700) of the *TSPYL5* gene (CpG islands, Fig. 3a) and sodium bisulfite-modified genomic DNA was used as a template. MSP results revealed a differential methylation pattern among the cells (Fig. 3b). *TSPYL5* gene exhibited decreased methylated band intensity in the following order: DU145 > LNCaP > RWPE-1. An intense methylation band was observed in DU145 (*P* =0.001) and LNCaP (*P* = 0.012) cells compared to RWPE-1 (Fig. 3c). While DU145 cells had no unmethylated band, the LNCaP cells had a very dim unmethylated band compared to RWPE-1 cells, which had an intermediate intensity unmethylated band.

**Fig. 4:**
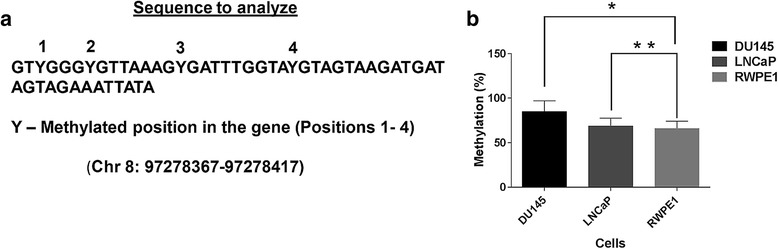
CpG methylation analysis by PSQ. **a** Analyzed sequence for methylation in *TSPYL5* promoter region in various cells. The cytosine residues are marked in *red* as “Y” and serially numbered. **b** Cumulative methylation of four cytosine residues in different cells. While methylation percentage was highest (1–1.2 fold; *P* = 0.04^*^) in DU145 cells relative to RWPE-1, the difference between LNCaP and RWPE-1 was insignificant (*P* > 0.05^**^)

In order to further investigate the extent of methylation in the cells, we analyzed a different chromosomal region within the *TSPYL5* gene (Chr 8: 97278367–97278417) for an individual cytosine methylation pattern using PSQ. PSQ quantifies methylation in explicit sequence context, thereby enabling several consecutive CpG sites to be quantified individually in a single assay. We selected the above region (CpG island shore, Fig. 3a) to avoid excessive CG density for design of PSQ primers. Four CpG sites were selected (Position 1–4, Fig. 4a and Additional file 1: Table S1) to analyze the extent of methylation in cytosine residues. Cumulative methylation percentage for individual cell type (Fig. 4b) indicated that the methylation percentage was highest (1.2 fold; *P* = 0.04) in DU145 cells relative to RWPE-1. The difference in methylation percentage of cytosine residues at the position of interest between LNCaP and RWPE-1 was not statistically significant (P > 0.05). The pyrograms depicting the methylation status of the cytosine residues in the selected sequence for DU145, LNCaP and RWPE-1 cells and the comparative methylation percentage of individual cytosine residues are shown in (Additional file 2: Figure S1).

### *TSPYL5* mRNA is expressed in human prostate tumor tissues and modulated by gene methylation

**Fig. 5:**
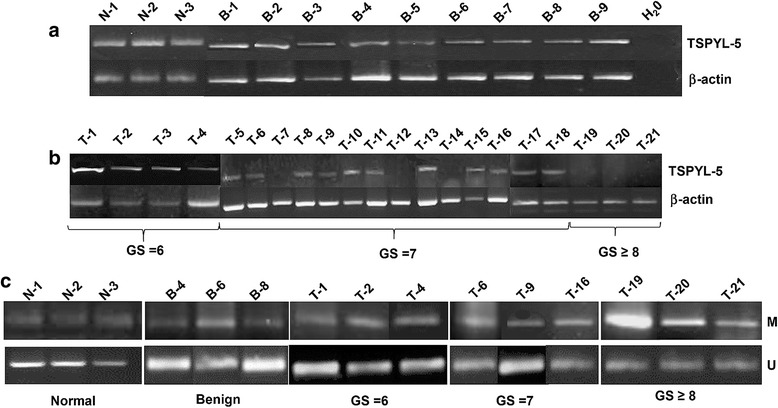
*TSPYL5* mRNA expression in tissues and MSP analysis. PCR analysis of *TSPYL5* mRNA expression in (**a**) benign, and **b** tumor tissues. Variable *TSPYL5* mRNA expression was observed in benign as well as tumor samples with insignificant expression in tumor tissues with GS ≥ 8. **c** A representative MSP results from benign and tumor samples (denoted above in the gels (**a** and **b**) by B# or T#). Low or moderate methylation alleles was observed in benign and tumor tissues with GS-6 or-7, while strong methylated bands was observed in tumor tissues with GS ≥ 8. Densitometry gel analysis indicated a decrease in the TSPYL5 mRNA expression in tissues with GS-7 (*P* = 0.012) and GS ≥ 8 (*P* = 0.001). PCR with unmethylated primers indicate the presence of unmethylated bands in benign and tumor tissues with GS-6 or −7, but relatively lower in tumor tissues with GS ≥ 8

After identifying an inverse relationship between *TSPYL5* mRNA and the presence of methylation in NT and PC cells, we sought to extend the analysis of gene expression and DNA methylation to benign and prostate tumor tissues. *TSPYL5* mRNA expression was observed in normal (*n* = 3) and benign samples (*n* = 9) (Fig. 5a). In total, 21 tumor samples were analyzed, out of which four samples had a GS of 6, fourteen samples had GS-7 and three samples had GS - 8 or- 9. Tumor tissues with GS ≥ 8 exhibited almost no *TSPYL5* expression (Fig. 5b, T19-21). Variable intermediate expression was observed with tumors with a GS-6 or −7 (T1-T18), however, in a few tumor GS-7 samples (T7, T12 and T14) very weak or no *TSPYL5* mRNA expression was observed. These three samples had Gleason pattern (4 + 3). The graphical representation of TSPYL5 mRNA expression in different tissues are depicted in (Additional file 3: Figure S2 (a)). Densitometry gel analysis indicated a decrease in the TSPYL5 mRNA expression in tissues with GS-7 (*P* = 0.012) and GS ≥ 8 (*P* = 0.001).

For MSP DNA methylation studies in tissues, the genomic DNA was isolated from normal, select benign and tumor samples (*n* = 3 each) and bisulfite converted before methylation analysis. MSP analysis with CpG island primers, demonstrate that methylation was low in normal and benign samples and lower or intermediate in graded tumor tissues (GS-6 or-7). Unfortunately, we had very little starting material of the tumor tissues with GS =7 (T7, T12, T14) were very little and were unable to assess the methylation analysis in these tissues. However in tissues with GS ≥ 8 increased methylation was observed (Fig. 5c). The cumulative methylation percentage is depicted in (Additional file 3: Figure S2 (b)). Significant methylation was observed between benign and tumor tissues (GS-7; *P* = 0.047) and GS ≥ 8 (*P* = 0.032). Furthermore, we analyzed the expression of DNMT’s in high grade advanced PC tumor tissues (Gleason score, GS ≥ 8) by IHC. It appears that predominantly DNMT3b protein was expressed while DNMT1 was found to be relatively low or not detected (Additional file 4: Figure S3).

### TSPYL5 protein expression in tumor tissues varies with advance grade

**Fig. 6:**
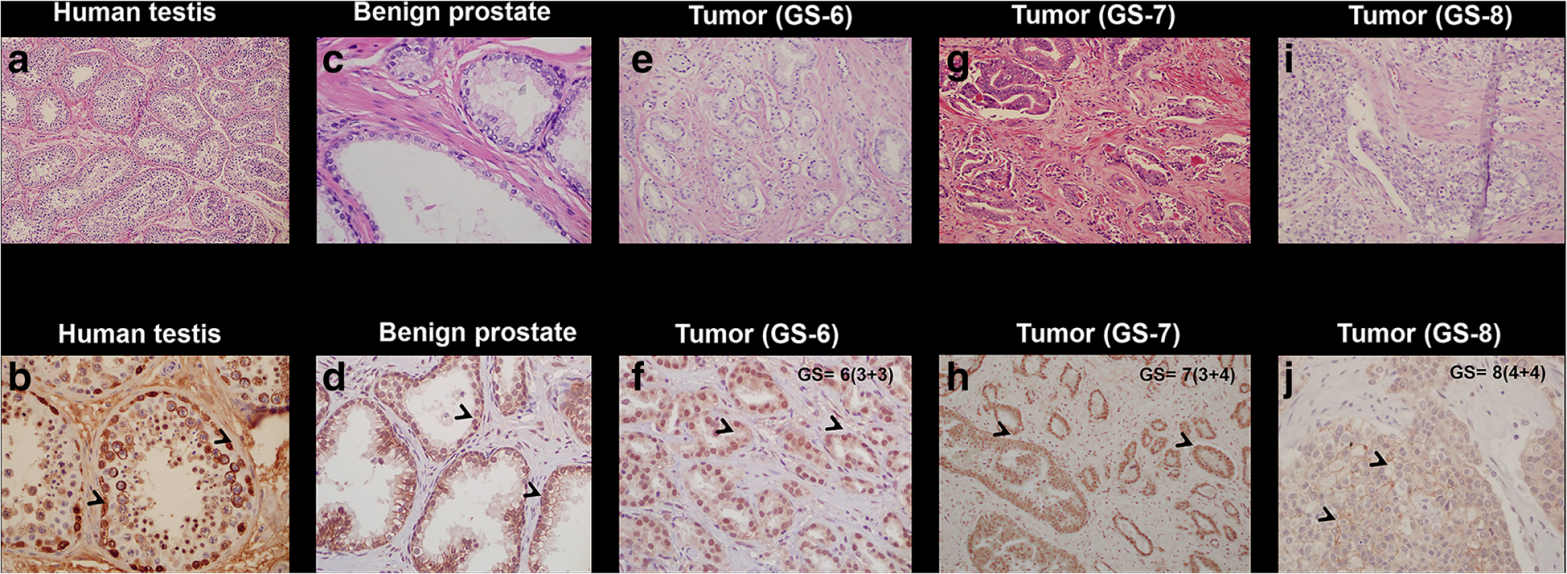
Immunohistochemical analysis in testis and benign prostate tissues for TSPYL5 protein expression. **a** Normal human testis showing seminiferous tubules with normal spermatogenesis (400×) (**b**) TSPYL5 protein expression in human testis tissue (positive control) which exhibited membrane, nuclear and cytoplasmic protein staining (400×). **c** Benign prostate tissues showing benign acini lined by inner secretory epithelial cells and outer basal cell layer (400×). **d** Benign prostate tissues exhibited more cytoplasmic membrane TSPYL5 protein staining (400×). **e** Prostate adenocarcinoma Gleason pattern 3 with small glands (400×). **f** Tumor tissues with GS-6 (3 + 3) exhibited both nuclear and cytoplasmic staining with a composite score (CS) of 12 (3x4) (400×). **g** Prostate adenocarcinoma Gleason pattern 4 with cribriform glands (400×). **h** Prostate adenocarcinoma with GS-7 (3 + 4), show moderate cytoplasmic and membrane staining with CS of 8 (2×4) (400×). **i** Prostate adenocarcinoma with Gleason score 4 (200×). **j** In the tumor specimens with GS-8 (4 + 4) or above the staining intensity was very low and confined to the membranes (CS = ≤1) with no nuclear or cytoplasmic staining (400×). The depicted images are representative of three cases examined individually in tissues with various GS. *Arrows* in the figure denotes TSPYL5 protein expression. (H & E-**a**, **c**, **e**, **g**, **i**; Immuno – **b**, **d**, **f**, **h**, **j**). The protein staining intensity between benign and tumor tissues with GS −8 (*P* = 0.012) and GS-7 (*P* = 0.032) were significant, while no significant difference was observed in staining intensities between benign and tumor tissues with GS-6 (*P* > 0.05)

IHC analysis of TSPYL5 in human normal prostate and tumor tissues identified protein expression patterns that mirrored the tissue mRNA expression data. A minimum of three tissues were analyzed in each case. Normal human testis tissue (Fig. 6a) was used as positive control for TSPYL5 expression. The testis tissue showed strong membrane, nuclear and cytoplasmic staining (Fig. 6b). In benign prostate tissue, benign acini lined by inner secretory epithelial cells and outer basal cell layer (Fig. 6c), the TSPYL5 expression was prominent in the cytoplasmic membrane (Fig. 6d). The prostate adenocarcinoma specimens with GS-6 (3 + 3), exhibited both nuclear and cytoplasmic staining with a composite score (CS) of 12 (3x4) (Fig. 6f). Prostate adenocarcinoma cases with GS-7 (3 + 4), show moderate cytoplasmic and membrane staining with CS of 8 (2x4) (Fig. 6h). Interestingly, in the tumor specimens with GS-8 (4 + 4) or above the staining intensity was very weak (CS ≤ 1) and is mainly confined to the cell membrane. There is a loss of nuclear and cytoplasmic staining (Fig. 6j). Corresponding H & E stains for testis, benign or prostate tumor tissues were processed in parallel (Fig. 6a, c, e, g and i). The composite scores obtained in tumor tissues with GS-6 or-7 were higher relative to the GS-8. The difference in protein staining intensity between benign and tumor tissues with GS −8 (*P* = 0.012) and GS-7 (*P* = 0.032) was significant, while no significant difference was observed in staining intensities between benign and tumor tissues with GS-6 (*P* > 0.05). Further analysis of tissues with GS 9 (4 + 5) compared to benign tissues (*P* = 0.008). (Additional file 5: Figure S4). Altogether, TSPYL5 expression diminishes in high grade prostate carcinoma compared to the benign tissue or intermediate grade prostate carcinoma suggesting that TSPYL5 could function as an indicator of disease progression. An overall summary of gene expression, methylation frequency and IHC composite scores are presented in Table 1. The patient tissues used in this study and their assigned Gleason scores are depicted in (Additional file 6: Table S2).

**Table 1:** Summary of *TSPYL5* mRNA expression, DNA methylation frequency, protein expression based on Gleason score

Pathology	Age (yrs)	GS	No of samples	mRNA^a^ expression	Methylation^b^ frequency (%)	IHC^c^
Normal	40–60	----	3	3/3	<10	-
Benign	59–79	----	9	7/9	25	-
Adenocarcinoma	36–50	6	4	3/4	28	12
Adenocarcinoma	50–70	7	14	11/14	39	8
Adenocarcinoma	60–68	≥8	3	0/3	70	0

^a^PCR analysis of *TSPYL5* mRNA expression in benign and prostate adenocarcinoma

^b^Analysis of DNA methylation (MSP) in the tissues (*n* = 3 each). The gel band intensities were quantified by Image J software as noted in methods

^c^Composite score (CS) is derived from staining intensity times percent positive cells for TSPYL5 protein expression

### TSPYL5 protein in cellular fractions and relative expression of other proteins

**Fig. 7:**
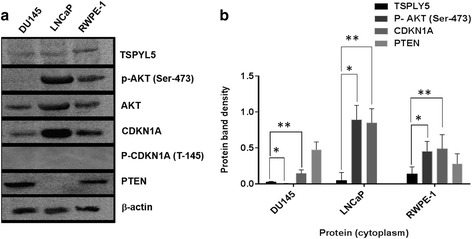
Immunoblot analysis for various protein expression in PC and non-tumorigenic cells. **a** Expression of TSPYL5 (~48 kDa), AKT (~61 kDA), pAKT (~61kDA), CDKN1A (~21 kDA), P-CDKN1A Thr-145 phosphorylated CDKN1A (~21kDA) and β-actin (~46 kDa). **b** Relative protein expression of TSPYL5, pAKT, CDKN1A and PTEN in the cells. Asterisk denotes respective protein (pAKT/TSPYL5* or CDKN1A/TSPYL5**) expression ratio based on the band intensity as described in the methods

We analyzed the expression of TSPYL5 and other proteins including CDKN1Aand pAKT in, DU145, LNCaP and RWPE-1 cells (Fig. 7a). The protein bands were analyzed (Fig. 7b) by image quantification software as described in the methods. TSPYL5 protein expression was absent in DU145, low in LNCaP and moderate in RWPE-1 cells.

To study the co-expression of other cellular proteins in all the cells, we focused on two important proteins CDKN1A and AKT which participate in cellular proliferation, drug sensitivity and cell survival. Interestingly, CDKN1A expression was low in DU145 cells in which TSPYL5 expression was insignificant. However, high CDKN1A expression was observed in LNCaP compared to RWPE-1 cells in which the endogenous TSPYL5 is either low or moderate, respectively. Phosphorylated CDKN1A (P- CDKN1A, Thr145) was absent in all cell lines. Variable AKT expression was observed in all these cells. Furthermore, LNCaP and RWPE-1 cells had pAKT expression, but no pAKT was observed in DU145. While PTEN (a tumor suppressor protein, TSP) is expressed in both DU145 and RWPE-1 cells in which TSPYL5 expression was absent or moderate, no expression was observed in LNCaP cells. Based on the protein band density (Fig. 7b), the ratios of pAKT/TSPYL5 in different cells were 0.25 (DU145), 18.9 (LNCaP) and 3.46 (RWPE-1). Also, the ratios between CDKN1A/TSPYL5 was 0.14 (DU145), 16.8 (LNCaP) and 3.7 (RWPE-1). These ratios indicate the differences in the relative expression of pAKT and CDKN1A in relation to TSPYL5.

### TSPYL5 overexpressing LNCaP cells exhibit enhanced sensitivity to chemotherapy drugs

**Fig. 8:**
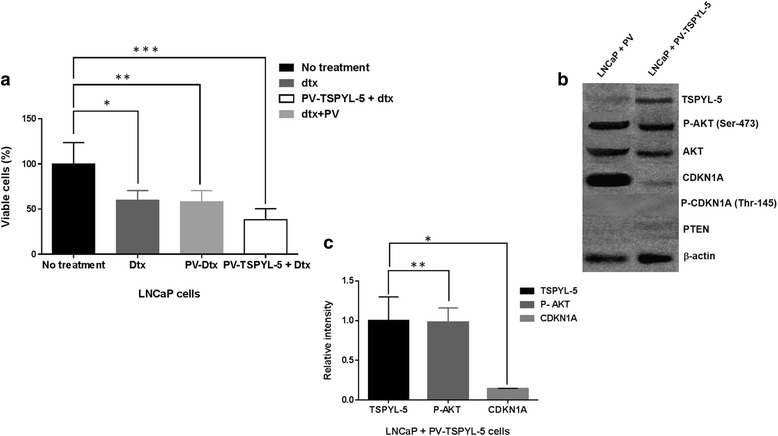
Drug sensitivity of TSPYL5 overexpressing LNCaP cells. **a** WT-LNCaP cells and PV-TSPYL5 or PV cells were treated with dtx (10 nM). Dtx caused reduction in cell viability of WT and PV only LNCaP cells (*P* = 0.012^*^, *P* = 0.014^**^). Dtx effect was more pronounced in cells treated with PV-TSPYL5 (*P* = 0.008^***^). **b** Various protein expression in PV-TSPYL5- and PV-LNCaP cells (**c**) Graph denoting relative protein band intensities in PV-TSPYL5 LNCaP cells between TSPYL5 protein, CDKN1A (*P* = 0.009^*^) and pAKT expression (*P* ≥ 0.05^**^)

In order to evaluate the drug sensitivity in WT and TSPYL5 overexpressing LNCaP cells, we tested the effect of two standard drugs used for PC treatment, paclitaxel (px) and docetaxel (dtx). Cellular viability in the presence of each drug (10 nM) was tested in WT, cells transfected with vector only (PV) and TSPYL5 overexpressing (PV-TSPYL5) LNCaP cells. Dtx decreased the WT and PV LNCaP cell viability by 40% (*P* = 0.012, *P* = 0.014). However, PV-TSPYL5 LNCaP cells exhibited enhanced sensitivity to dtx (62%, *P* = 0.008) compared to WT cells without the drug (Fig. 8a). Similar results were observed in the presence of px (Additional file 7: Figure S5). Changes in pAKT, CDKN1A and PTEN protein expression in TSPYL5 overexpressing LNCaP cells were analyzed by Western analysis. We noted a reduction in CDKN1A (*P* = 0.009) in PV-TSPYL5 LNCaP cells which originally had high endogenous CDKN1A. Only a mild decrease (P ≥ 0.05) was observed with pAKT and no changes in PTEN expression (Fig. 8b and c).

## Discussion

In this study we have demonstrated that the presence of DNA methylation in the 5′ region of the gene is negatively associated with expression of *TSPYL5* mRNA and protein in PC cells, NT cells and clinical prostate tissue samples. Methylation induced TSPYL5 gene silencing was previously reported in glioma and gastric cancer types [15, 16]. The TSPYL5 protein expression mirrored the expression pattern of mRNA in the cells (DU145, LNCaP or RWPE-1). The TSPYL proteins are members of the NAP superfamily of proteins [9] that have been shown to bind to proteins involved in transcription, cell cycle regulation [25], and shuttling histones between nucleus and cytoplasm [26]. However, it is not clear whether such a function for TSPYL5 exists in PC cells.

Previous studies with colorectal HCT116 cells indicated that both DNMT1 and DNMT3B enzymes were essential to methylate *TSPYL5* gene promoter regions [15]. While one or the other enzyme was observed in the cells tested in this study, we observed only DNMT3B protein was predominantly expressed in more advanced PC tissues in which TSPYL5 was absent. Earlier studies in prostate cancer have analyzed various methyltransferases and found that DNMT1 expression was found to be lower than DNMT3b. Further, de novo methylation remains in DNMT1 knockout embryonic stem cells and the role of DNMT1 in tumor methylation remains ambiguous [27]. Depending on the cellular context, the TSPYL5 gene might be differentially targeted for methylation by methyltransferases.

Previous studies have shown a correlation between methylation in chromosome 8 region (Chr 8: 97278129–97278175) and loss of *TSPYL5* gene expression in lung carcinoma cells, although, no tissue studies or normal cell studies have been done [17]. While CpG islands are important to regulate gene expression [28], previous studies suggest that the lower density CpG shores of islands may also be important [29]. Our studies with MSP analysis of the CpG island identified methylation of the *TSPYL5* gene in PC cells and tissues. As anticipated, PSQ analysis of CpG dinucleotides on the 5′ shore of the CpG island revealed higher methylation of the four cytosine residues (Chr 8: 97278367–97278417) in DU145 cells relative to other cell lines. Only a subtle difference was observed in individual cytosine methylation between, LNCaP and RWPE-1. This is in keeping with our observations that DU145 had the least *TSPYL5* expression due to methylation-induced gene silencing.

Planning treatment for prostate cancer patients relies on histopathological grading by GS [30] which currently lacks a precise molecular correlate [6, 7]. There is a critical need to identify companion biomoleules that distinguish more advanced phenotype tumors within intermediate GS-7 specimens. Our studies identified *TSPYL5* mRNA and protein expression in benign and tumor tissues with a GS-6 or-7. High grade tumors with GS ≥ 8 had the least expression of TSPYL5, likely due to DNA methylation. Interestingly, a few GS-7 tumor samples with Gleason pattern (4 + 3) had no message for *TSPYL5*. At this time, it is not clear whether the absence of *TSPYL5* mRNA expression in tissues with GS 7 (4 + 3) would indicate any undetected higher-grade disease. Further studies with more tissues are needed to assess this possibility. Taken together, these data suggest that the absence of TSPYL5 may be an indicator of more advanced prostate cancer disease.

MSP analysis of the *TSPYL5* gene indicated a mixture of methylated and unmethylated bands in benign and intermediate-grade tumors with GS-6 or −7, while GS-8 tumors had predominantly methylated bands suggesting a methylation induced *TSPYL5* silencing in these tumors. Previous studies indicated that *TSPYL5* could be an independent marker of poor outcome in breast cancer based on their high expression in aggressive basal-like breast cancers [31]. On the contrary, we observed both by mRNA expression and IHC that TSPYL5 expression diminishes in high grade tumors. Such a difference in TSPYL5 expression could be exploited to identify the clinical behavior of intermediate grade prostate tumors (GS-7). A recent report suggested the use of higher levels of SNPs-rs2735839 to stratify patients with GS-7 because of the association with aggressive PC [32]. However, to classify GS-7 patients based on diminished TSPYL5, large cohorts of prostate tumor samples will need to be investigated. Studies along this direction are in progress in our laboratory.

In addition to its anti-proliferative role, CDKN1A is also vital to proliferation and survival. A previous study reported that knockdown of *TSPYL5* increased the endogenous expression of p53 and its downstream target CDKN1A in MCF7 breast carcinoma cells [31]. It has been reported that in lung carcinoma cells, TSPYL5 was able to suppress CDKN1A by modulating PTEN/AKT pathway [17]. Also, *TSPYL5* gene silencing increased the CDKN1A protein expression and caused growth reduction in cells [17]. However, we observed that TSPYL5 gene silenced cells (DU145) exhibited very minimal expression of CDKN1A. Conversely, low or moderately TSPYL5 expressing LNCaP and RWPE-1 cells showed high and relatively low CDKN1A expression. We also observed a decrease in CDKN1Aprotein expression in TSPYL5 overexpressing LNCaP cells. However, in contrast to lung carcinoma cells [17] LNCaP cells lack *PTEN*, so any involvement of TSPYL5 in modulating CDKN1A must work by a PTEN-independent mechanism [33].

Our observations identified that even low *TSPYL5* expressing cells (eg: LNCaP) had higher pAKT. Also, TSPYL5 expressing RWPE-1 cells expressed basal pAKT, albeit low levels compared to LNCaP cells. This in sharp contrast to the observation made in lung carcinoma cells [17] that high TSPLY-5 expression can activate AKT. The exact role of *TSPYL5* is not clear in modulating AKT expression in PC cells and could vary depending on the cellular phenotype.

Mounting evidence suggests that TSG’s play an important role in the response to a variety of chemotherapeutic drugs such as px, 5-Fluorouracil, cisplatin and trastuzumab [34, 35]. It was reported that a decrease in retinoblastoma (Rb) protein, a TSP, in sarcoma cells conferred resistance to doxorubicin and cisplatin [36]. PC cells and glioblastoma cells deficient in Rb were resistant to cisplatin and doxorubicin, respectively [37, 38]. Similarly, p53 inactivation resulted in reduced sensitivity to cisplatin but not px in ovarian carcinoma cells, suggesting that the role of p53 in response to chemotherapy depends on both cellular context as well as the class of chemotherapeutic compounds [39]. Increased expression of CDKN1A leads to chemoresistance, and its loss sensitizes the cells to chemotherapy response [40, 41]. Interestingly, it was reported earlier that LNCaP cells were resistant for dtx and knockdown of p53 protein increases its sensitivity to the drug by decreasing CDKN1A [42]. Previous studies had shown that increase in TSPYL5 can compete with USP7, a deubiqutinylating protein thereby decreasing p53 in MCF 7 cells [31]. Our studies show that reduction in CDKN1A in TSPYL5 overexpressing LNCaP cells exhibit more sensitivity for dtx and px compared to WT cells. All the above studies highlight the possible roles of TSP’s in chemotherapy response. Our studies suggest that increased TSPYL5 enhances the sensitivity of the cells to chemotherapy drugs, likely by downregulating CDKN1A. While it is tempting to suggest that *TSPYL5* status in PC cells could be indicative of predicting chemotherapy response, further studies are needed to substantiate this notion. In keeping with the previous studies regarding the role of TSP’s in chemosensitivity [40, 41], we speculate that the response to chemotherapy drugs in *TSPYL5* expressing PC cells likely may vary depending upon the cellular context and the type of chemotherapy drug.

## Conclusions

In conclusion, our studies demonstrate the potential significance of *TSPYL5* in prostate carcinoma and that it could have more than one function depending on the cellular phenotype. Methylation of this gene in tumor tissues correlated inversely with its mRNA and protein expression. Reduced gene product in high GS tumors suggests that TSPYL5 could likely function as an indicator of more advanced prostate carcinoma. Absence of *TSPYL5* in the PC cells could influence the expression of pleotropic proteins such as CDKN1A, which has been implicated in pro-proliferative, survival and anti-apoptotic roles. Further, the absence of *TSPYL5* in PC cells may have consequences similar to other canonical TSP’s such as PTEN or Rb in becoming resistant to chemotherapy drug treatment.

## Additional files

Additional file 1: Table S1 Original and bisulfite converted sequence of *TSPYL5* gene region in chromosome 8 for PSQ Analysis.
Additional file 2: Figure S1. Methylation analysis by pyrosequencing. (a) Pyrograms depicting the methylation of individual cytosine residues in DU145, LNCaP and RWPE-1 cells. (b) Graph depicting the percentage methylation of four bases across different cells. (TIF 1465 kb)
Additional file 3: Figure S2.
*TSPYL5* mRNA expression and methylation in normal and prostate tumor tissues. TSPYL5 mRNA expression in benign and prostate tissues after PCR and subsequent gel analysis (Fig. 5a and b). The results were analyzed as described in methods and depicted as cumulative percent expression (b) MSP analysis of benign and prostate tumor tissues (Fig. 5c). Gel bands were analyzed for individual samples, and average in each group was obtained and depicted as cumulative percent methylation as described in methods. (TIF 118 kb)
Additional file 4: Figure S3. IHC analysis for DNMT3b (a and b) and DNMT1 (c and d) in prostate tumor (GS = > 8) tissues. (a) Tumor tissue (No Ab control) (20×). (b) Tumor tissue positive for DNMT3b protein in the nucleus (arrows) treated with DNMT3b Ab (40×). (c) Tumor tissue (No Ab control) (20×). (d) Tumor tissue treated with DNMT-1 Ab is negative for the protein (40×). (TIF 3624 kb)
Additional file 5: Figure S4. IHC analysis for TSPYL5 protein in high grade (GS = 9 (4 + 5)) prostate tumor tissue. (a) H & E stain for tumor tissue. (b) Tumor tissue treated with TSPYL5 antibody is negative for the protein. Magnification (400×). (TIF 2038 kb)
Additional file 6: Table S2 Patient tumor tissues and Gleason scores.
Additional file 7: Figure S5. Effect of px on LNCaP cells. WT, PV and PV-TSPYL-5 LNCaP cells were exposed to 10 nM px. While px decreased the viability of LNCaP cells per se, the effect was more pronounced in PV-TSPYL-5 cells. (TIF 75 kb)

## Abbreviations

dtx: Docetaxel
GS: Gleason score
IHC: Immunohistochemistry
MSP: Methyl-specific PCR
NT: Non-tumor
PC: Prostate carcinoma
PSQ: Pyrosequencing
px: Paclitaxel
TSG: Tumor suppressor gene
TSP: Tumor suppressor protein
TSPYL5: Testis specific Y-like 5
WT: Wild type
5-AzadCyD: 5-aza-2′-deoxycytidine
