# Supplementary figures and images for: Testis specific Y-like 5: gene expression, methylation and implications for drug sensitivity in prostate carcinoma
Source: BMC Cancer. 2017 Feb 24;17:158. doi: 10.1186/s12885-017-3134-7 (PMC5326500; doi:10.1186/s12885-017-3134-7)

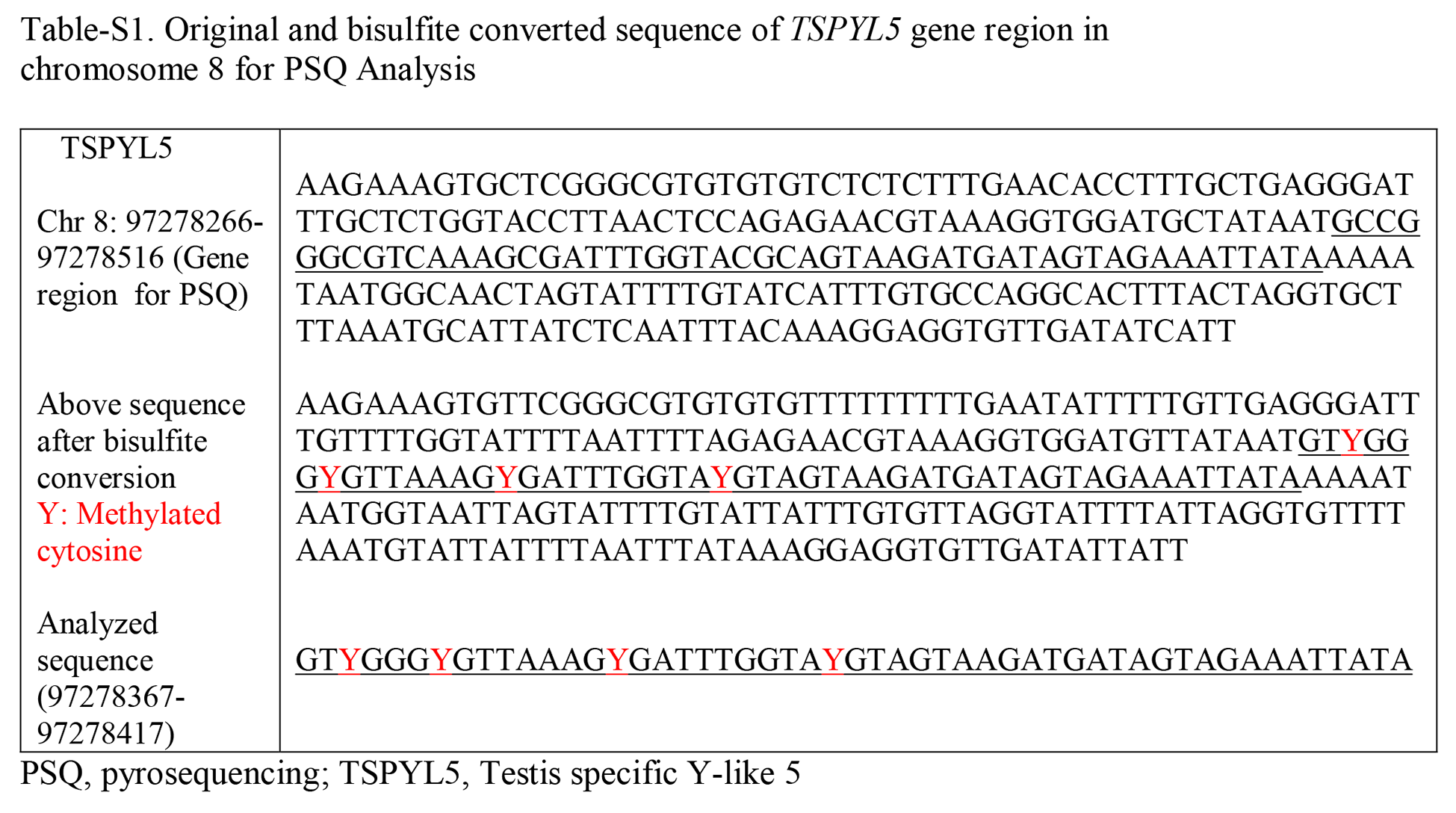

Supplement: Additional file 1: Table S1 — Original and bisulfite converted sequence of TSPYL5 gene region in chromosome 8 for PSQ Analysis. [file 12885_2017_3134_MOESM1_ESM.tif]

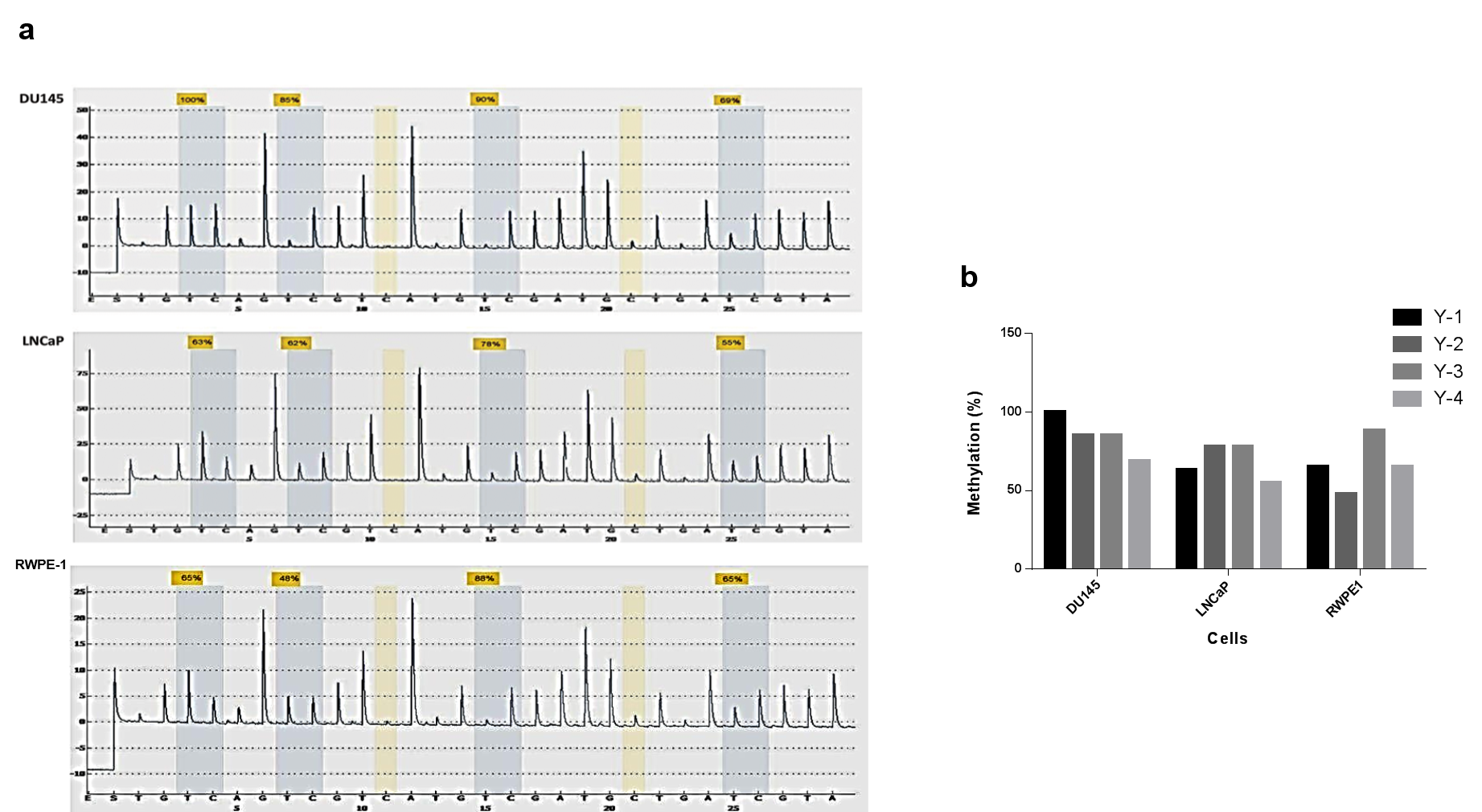

Supplement: Additional file 2: Figure S1. — Methylation analysis by pyrosequencing. (a) Pyrograms depicting the methylation of individual cytosine residues in DU145, LNCaP and RWPE-1 cells. (b) Graph depicting the percentage methylation of four bases across different cells. (TIF 1465 kb) [file 12885_2017_3134_MOESM2_ESM.tif]

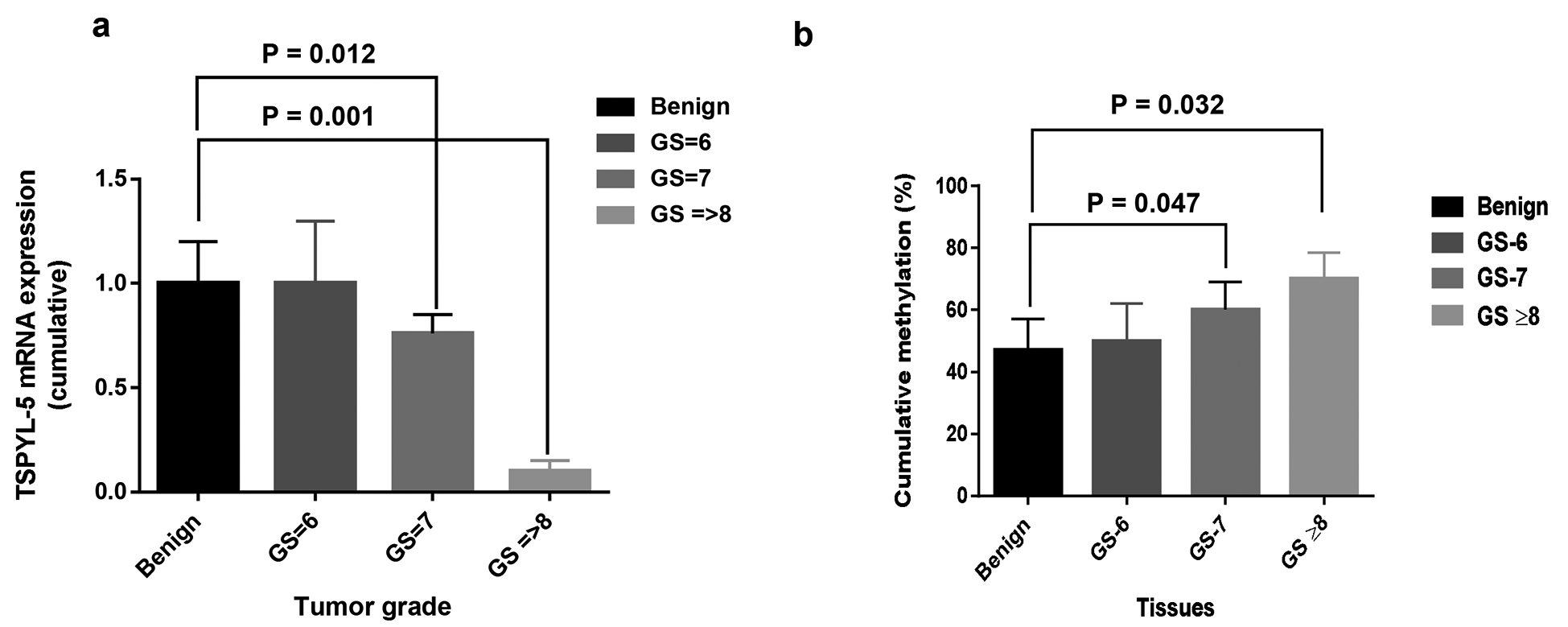

Supplement: Additional file 3: Figure S2. — TSPYL5 mRNA expression and methylation in normal and prostate tumor tissues. TSPYL5 mRNA expression in benign and prostate tissues after PCR and subsequent gel analysis (Fig. 5a and b). The results were analyzed as described in methods and depicted as cumulative percent expression (b) MSP analysis of benign and prostate tumor tissues (Fig. 5c). Gel bands were analyzed for individual samples, and average in each group was obtained and depicted as cumulative percent methylation as described in methods. (TIF 118 kb) [file 12885_2017_3134_MOESM3_ESM.tif]

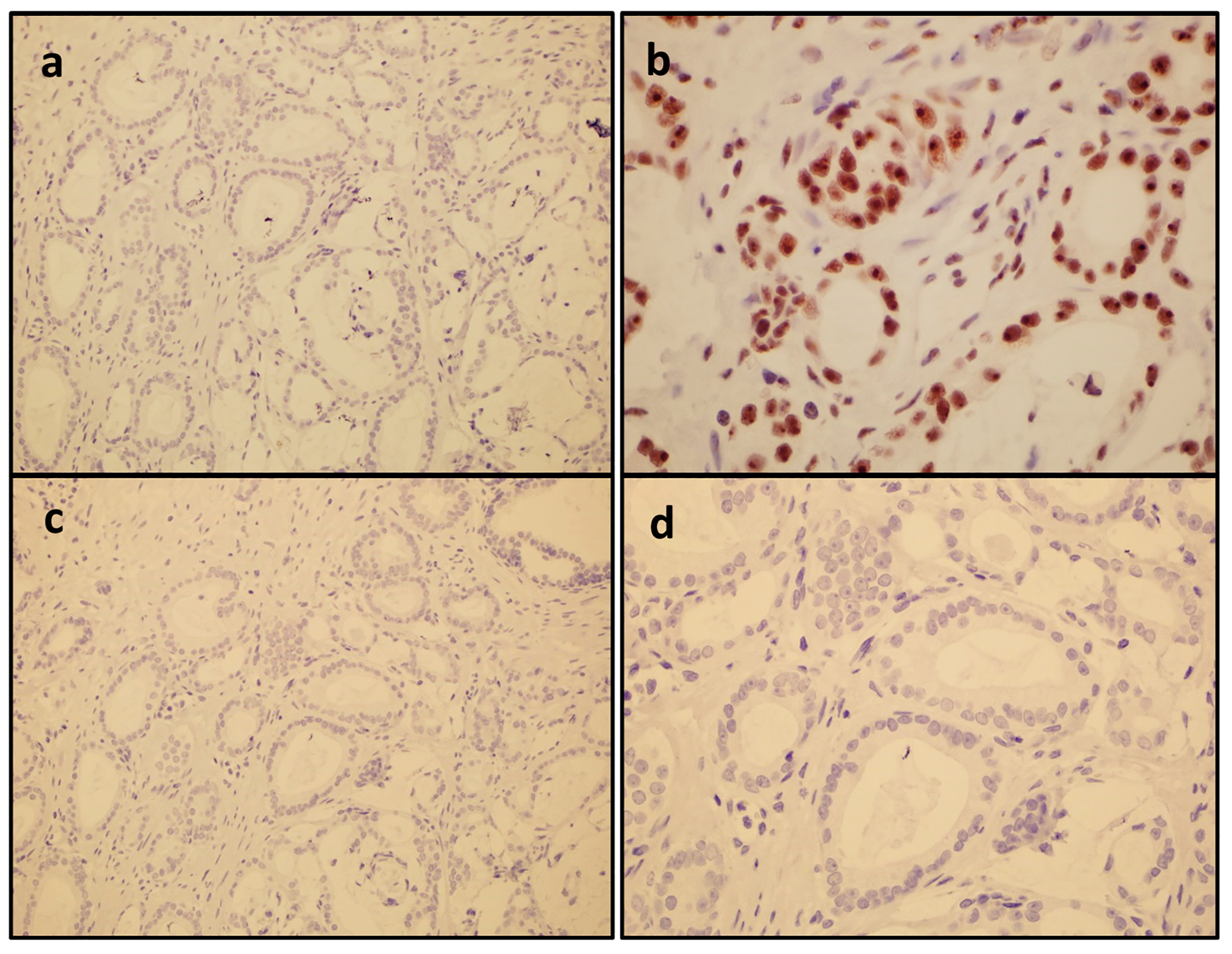

Supplement: Additional file 4: Figure S3. — IHC analysis for DNMT3b (a and b) and DNMT1 (c and d) in prostate tumor (GS = > 8) tissues. (a) Tumor tissue (No Ab control) (20×). (b) Tumor tissue positive for DNMT3b protein in the nucleus (arrows) treated with DNMT3b Ab (40×). (c) Tumor tissue (No Ab control) (20×). (d) Tumor tissue treated with DNMT-1 Ab is negative for the protein (40×). (TIF 3624 kb) [file 12885_2017_3134_MOESM4_ESM.tif]

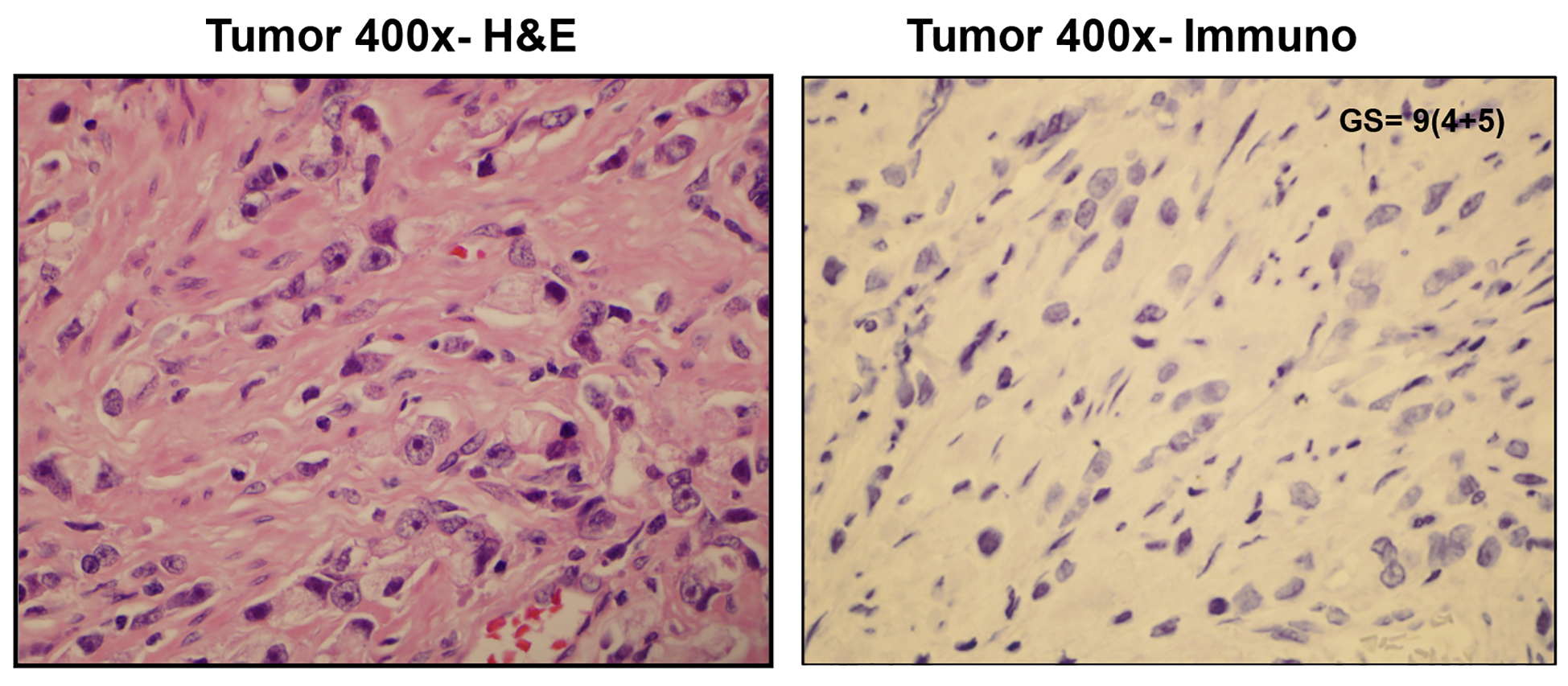

Supplement: Additional file 5: Figure S4. — IHC analysis for TSPYL5 protein in high grade (GS = 9 (4 + 5)) prostate tumor tissue. (a) H & E stain for tumor tissue. (b) Tumor tissue treated with TSPYL5 antibody is negative for the protein. Magnification (400×). (TIF 2038 kb) [file 12885_2017_3134_MOESM5_ESM.tif]

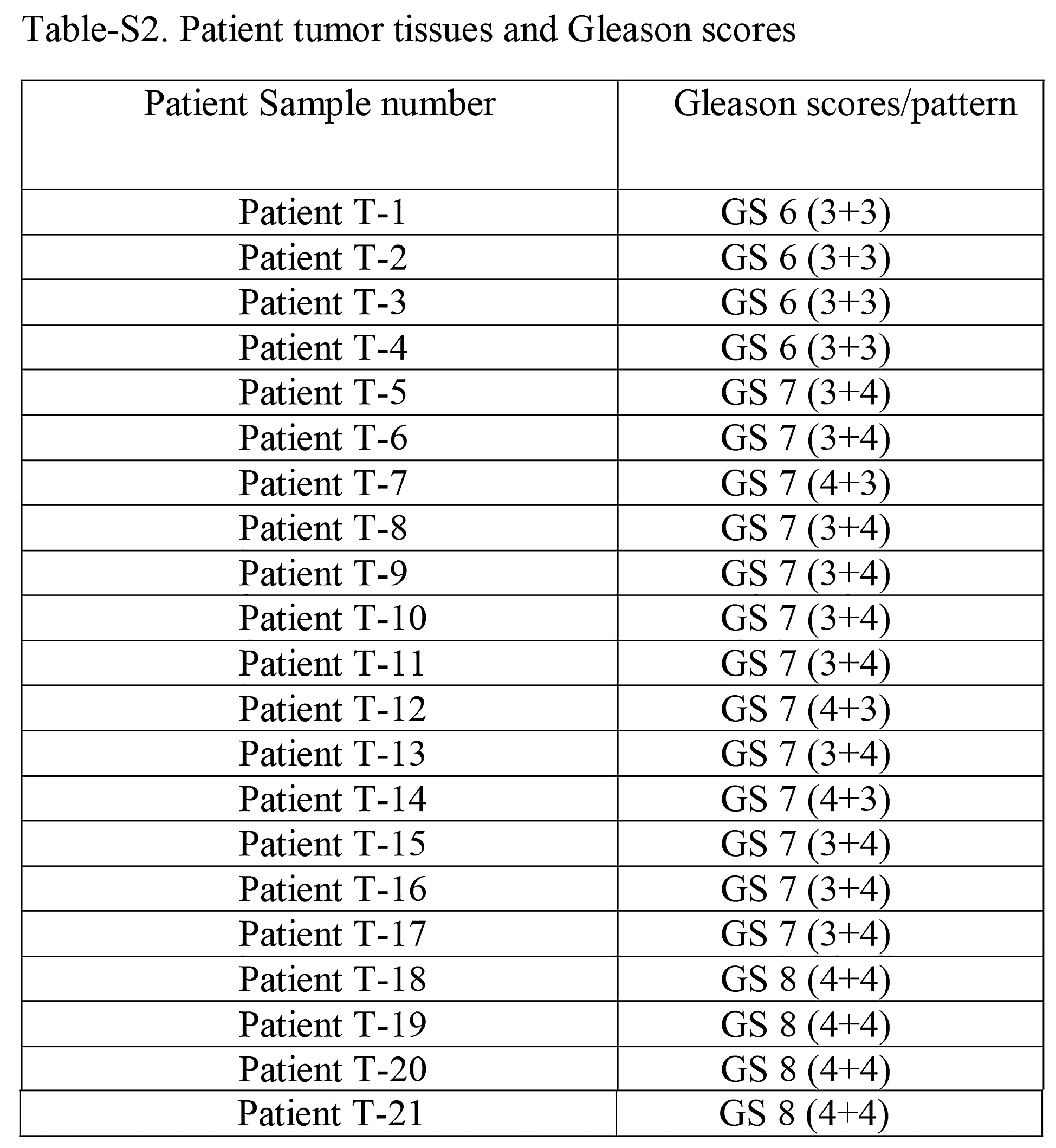

Supplement: Additional file 6: Table S2 — Patient tumor tissues and Gleason scores. [file 12885_2017_3134_MOESM6_ESM.tif]

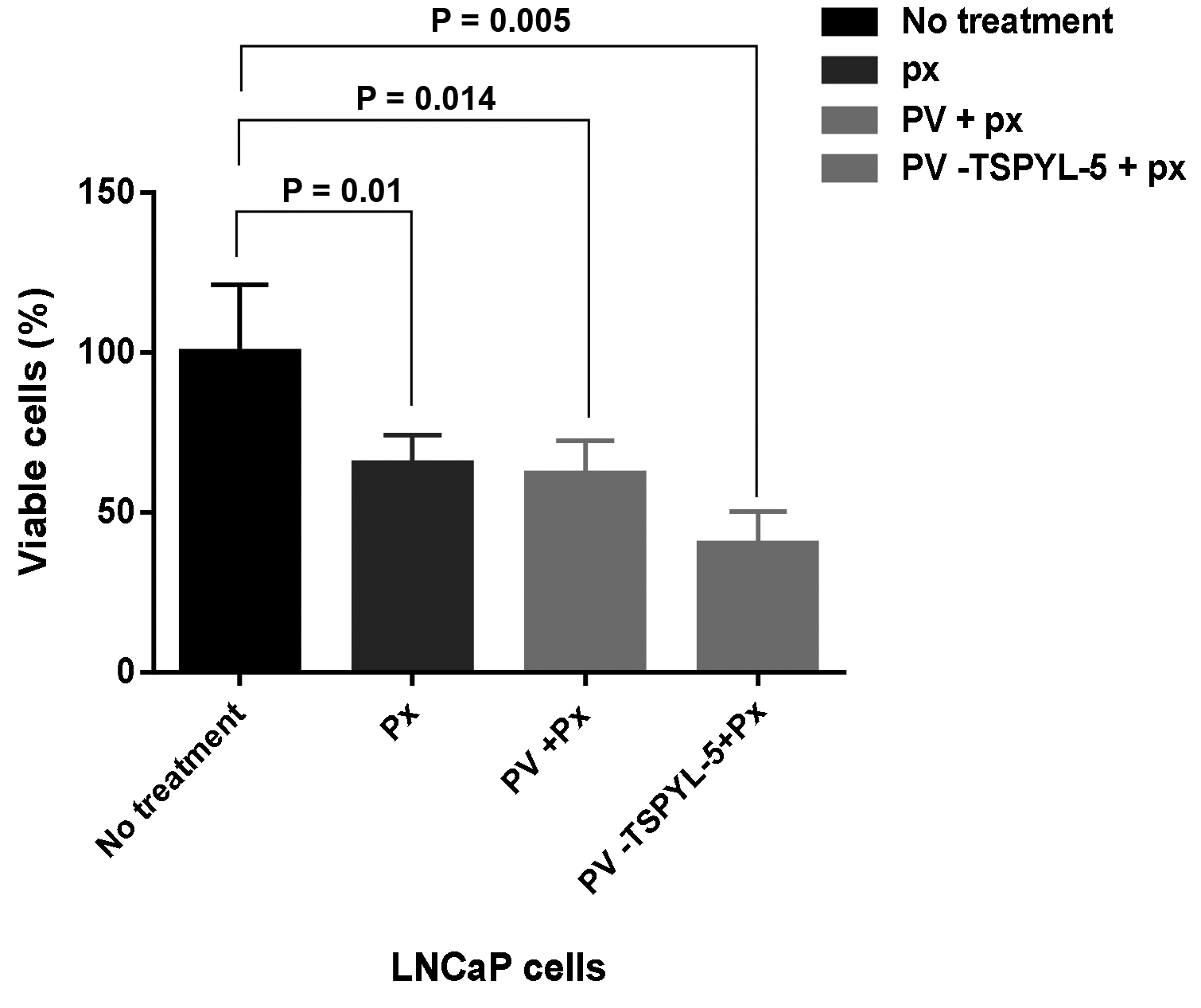

Supplement: Additional file 7: Figure S5. — Effect of px on LNCaP cells. WT, PV and PV-TSPYL-5 LNCaP cells were exposed to 10 nM px. While px decreased the viability of LNCaP cells per se, the effect was more pronounced in PV-TSPYL-5 cells. (TIF 75 kb) [file 12885_2017_3134_MOESM7_ESM.tif]
